# A Retrospective Analysis of 129 Ameloblastoma Cases: Clinical and Demographical Trends from a Single Institution

**DOI:** 10.1007/s40615-024-01993-3

**Published:** 2024-04-12

**Authors:** Stefan Vila, Robert A. Oster, Sherin James, Anthony B. Morlandt, Kathlyn K. Powell, Hope M. Amm

**Affiliations:** 1 Department of Oral & Maxillofacial Surgery, University of Alabama at Birmingham, Birmingham, USA; 2 Department of Medicine, University of Alabama at Birmingham, Birmingham, USA; 3 Department of Otolaryngology, University of Alabama at Birmingham, Birmingham, USA

**Keywords:** Ameloblastoma, Race, Ethnicity, Oral Surgery

## Abstract

Ameloblastomas are benign neoplasms of the jaw, but frequently require extensive surgery. The aim of the study was to analyze the demographic and clinicopathological features of ameloblastoma cases at a single Oral and Maxillofacial Surgery group in the United States. Study Design: A retrospective chart review of patients evaluated for ameloblastoma between 2010 and 2020 at a single tertiary care center. Age, race, sex, tumor size, tumor location, and histological subtypes were recorded. Results: A total of 129 cases of ameloblastoma were recorded with a mean patient age of 42 ± 18.6 years (range 9–91 years old), male to female ratio 1.08:1. Ameloblastoma presenting in the mandible outnumbered maxilla in primary (118 to 8, respectively) and recurrent cases (8 to 1, respectively). There was a higher prevalence of ameloblastoma in Black patients (61.3%) with mean age of Black patients occurring at 40.5 years and the mean age of White patients occurring at 47.8 years and mean tumor size trended larger in the Black patients (15.7 cm^2^) compared to White patients (11.8 cm^2^). Conclusion: Data suggests a strong influence of racial factors on the incidence of ameloblastoma, with regards to size, Black patients with ameloblastoma trended higher and more data is needed to clearly elucidate any relationship between the tumor size and race, as other factors may influence the size (such as time to discovery).

**Supplementary Information:**

The online version contains supplementary material available at 10.1007/s40615-024-01993-3.

## Introduction

Ameloblastomas are one of the most common of odontogenic tumors that occur within the bones of the jaw, primarily in the posterior mandible [1]. Ameloblastomas are estimated to be approximately 1% of all cysts, less than 1% of head and neck tumors, and 11 to 59% of odontogenic tumors, depending on the report and geographical location [2–5]. The World Health Organization (WHO) classifies ameloblastomas as five types: conventional ameloblastoma (previously known as multicystic or solid), unicystic ameloblastoma (6%), extraosseous/peripheral ameloblastoma (2%), and metastasizing ameloblastoma (1%), and adenoid ameloblastoma, a type designated in 2022 [6, 7]. Though they are classified as benign and generally slow growing, ameloblastomas are locally invasive and destructive, and in rare cases metastasize to other organs similar to malignant tumors.^2^ The exact origin and pathogenesis of these neoplasms is unknown but is believed to arise from the enamel organ of developing teeth, epithelium of other odontogenic cysts, or the stratified squamous epithelium [8, 9]. More recent understanding of the biological factors shows that ameloblastomas arising from the mandible are likely to be associated with mutations in the MAPK pathway, with BRAFV600E mutations being the most common, while those arising from the maxilla tend to have SMO mutations [10–13]. These mutations may lead to non-surgical targeted treatment options through targeted therapy [10–14].

Currently, surgical excision is the primary therapy for ameloblastomas with an overall recurrence rate of approximately 30%; however, recurrence rates are higher with more conservative surgical treatment [1, 3, 15, 16]. The growth can begin developing asymptomatically, with most presentations appearing in the fourth and fifth decades of life [1]. Symptoms most often present as regional swelling, but pain and nasal obstruction can occur [13, 14]. Though the prognosis is often positive when treated early, an untreated ameloblastoma can lead to jaw damage, facial deformity, and even death due to complications from rare metastases, infection, and damage to critical structures in the head and neck [15].

There have been both single and multiple site international retrospective studies, such as in Asia and Africa but limited US or North American data [16–20]. Amongst those studies, Black patients have incidence rates of ameloblastoma five times more than Whites in North and South American populations and Black patients have more aggressive tumors. The purpose of this retrospective investigation is a comprehensive study of ameloblastoma cases in the Southeastern region of the United States. This region is uniquely suited for this study as Black patients are reported to have a higher risk of developing ameloblastoma, and an estimated 55% of Black Americans live in this region (U.S. Census) [21, 22]. We hypothesize that our patient population will have a larger percentage of Black compared to White patients and that black patients will have larger tumors. Predominance of male versus female patients with ameloblastoma have been mixed, some studies show a predominance in one sex and others have not. Few studies have considered tumor size in their comparisons; therefore, we did not know if sex was related to tumor size. A total of 129 cases were collected across 10 years, composed of varied demographics in age and race. An analysis of patient age and tumor size of ameloblastoma at time of treatment was conducted to determine if age or tumor size correlated with race. Here we report that although Black patients presented with ameloblastoma at a significantly younger age these patients tended to have larger tumors.

## Materials and Methods

### Study Design

This retrospective chart review was conducted at a single tertiary care center. The study was approved by the University of Alabama at Birmingham Institutional Review Board (IRB #300001887) and followed all the federal guidelines in accordance with the World Medical Association Declaration of Helsinki. A total of 614 subjects were identified using the Internal Classification of Disease (ICD-O-3) codes 213.1 and D16.5 (benign neoplasm of jawbone), with 129 of these cases presenting as ameloblastoma. Confirmed cases were divided into three subtypes: conventional/solid/multicystic, unicystic, and extraosseous/peripheral ameloblastomas [3, 23]. Conventional tumors were subdivided based on histopathologic features: follicular, plexiform, acanthomatous, desmoplastic, granular cell, or basal cell pattern. Unicystic tumors were subdivided into three histopathological subtypes: luminal, intraluminal/plexiform, or mural pattern [3, 24].

Subjects’ ages ranged between 9 and 91 at time of treatment or evaluation at UAB. Subjects’ race was self-reported as White, Black, Asian, Hispanic, American Indian, or Alaskan Eskimo. Patients with no race reported were labeled as unknown.

### Data Collection

All electronic medical records were collected from the UAB Cerner electronic health records and the subjects’ demographic details (including race, age), tumor site (mandible or maxilla), histological subtype, as well as tumor size were recorded. Histological subtype was collected from a combination of biopsy and surgical specimen pathology reports, and tumor size from surgical specimen measurements and pre-operative computed tomography scans.

### Data Analysis

Descriptive statistics were obtained for all study variables. Means of age and tumor size (cm^2^) were compared using the two-group t-test. Correlation coefficients were obtained and tested using Pearson correlation analysis (or Spearman correlation analysis when at least one of the variables was categorical). Multiple variable analyses were performed using multiple variable linear regression analysis. The distributions of age and tumor size were examined for normality using graphical techniques and statistical tests. Since tumor size was not normally distributed, we log_10_ transformed this prior to statistical analysis (the log transformed tumor size values are normally distributed). All analyses were performed using SAS, version 9.4 (SAS Institute, Cary, NC).

## Results

### Patient Demographics

A total of 129 ameloblastoma patient cases were identified over the decade [2010] to [2020]. This accounted for 21.0% of cases identified using the Internal Classification of Disease (ICD-O-3) codes 213.1 and D16.5 (benign neoplasm of jawbone). Among these 129 patients, 9 of them were confirmed with the recurrence of ameloblastoma. The male to female patient ratio was 67 to 62 (1.08:1). The mean age across all patients was 42.3 (± 18.7) years with a range of 9 to 91 years. The ages between male (42.9 ± 17.7) and female (41.6 ± 19.9) patients were not statistically different, with *p* = 0.717 (Table 1). Among the 129 patients, there were 77 Black patients (61.2%), 44 White patients (34.1%), 3 Asian (2.3%), 1 Hispanic, 1 American Indian, and 1 of unknown/unreported race (0.78%).

**Table 1:** Race and sex distribution

Race	All [*n* %)]	Primary [*n* %)]	Recurrent [*n* %)]
**White**	44 (34.1)	39 (32.5)	5 (55.6)
Male	27 (20.9)	26 (21.7)	1 (11.1)
Female	14 (10.9)	13 (10.8)	4 (44.4)
**Black**	79 (61.2)	76 (63.3)	3 (33.3)
Male	36 (27.9)	36 (30.0)	−
Female	40 (31.0)	40 (33.3)	3 (33.3)
**Asian**	3 (2.3)	3 (2.5)	−
Male	3 (2.3)	3 (2.5)	−
Female	−	−	−
**Hispanic**	1 (0.8)	1 (0.83)	−
Male	−	−	−
Female	1 (0.8)	1 (0.83)	−
**American Indian**	1 (0.8)	1 (0.83)	−
Male	−	−	−
Female	1 (0.8)	1 (0.83)	−
**Unknown**	1 (0.8)	−	1 (11.1)
Male	1 (0.8)	−	−
Female	−	−	−
**Total**	129	120 (100)	9 (100)

^[*n* (%)] = number of patients (% of patients per population: all, primary, and recurrent)^

### Tumor Site and Type

The tumor sites were predominantly found in the mandible (93%), with a small portion found in the maxilla (7%) (Table 2). Among the patients, 97 were diagnosed with conventional ameloblastomas (75.2%), 24 with unicystic ameloblastoma (18.6%), and 1 with peripheral ameloblastoma (0.78%). For 4 patients (3.1%), the type of ameloblastoma was not recorded or available based on clinical data. The mean age for patients with unicystic ameloblastomas (33.5 ± 12.1) was significantly lower than those with conventional ameloblastoma (43.1 ± 17.6).

**Table 2:** Location and type of ameloblastoma

	All [*n* (%)]	Primary [*n* (%)]	Recurrent [*n* (%)]
**Mandible**	120 (93.0)		
Conventional	91 (70.5)	83 (91.2)	8 (8.8)
Unicystic	24 (18.6)	21 (87.5)	3 (12.5)
Peripheral	1 (0.8)	1 (100)	0 (0)
Unknown	4 (3.1)	4 (100)	0 (0)
**Maxilla**	9 (7.0)		
Conventional	9 (7.0)	8 (88.9)	1 (11.1)
Unicystic	0 (0)	0 (0)	0 (0)
Peripheral	0 (0)	0 (0)	0 (0)
**Total**	129	120 (93.0)	9 (7.0)

^[*n* (%)] = number of patients (% of total patients)^

### Histological Subtype

**Fig. 1:**
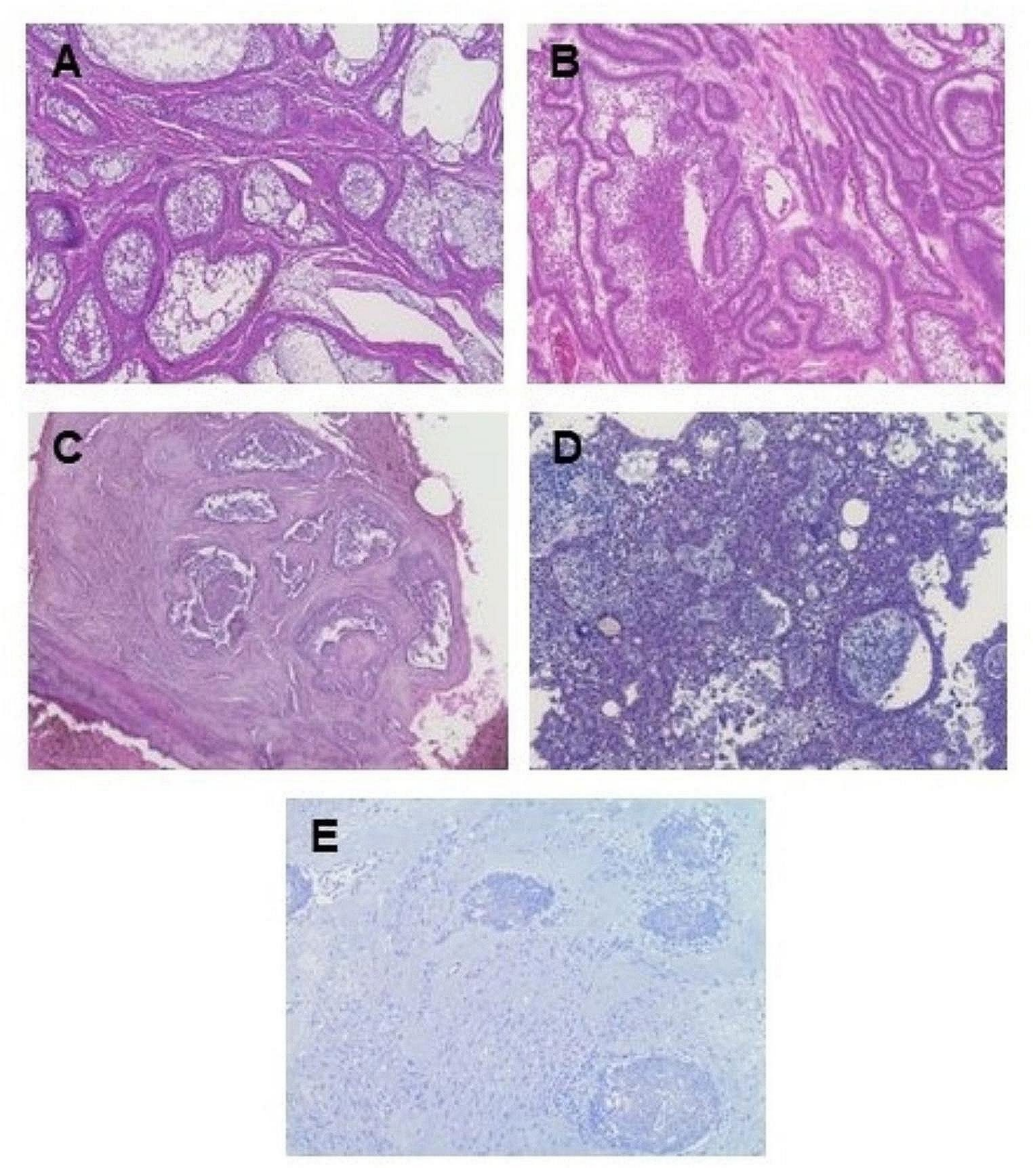
Ameloblastoma histological patterns, H&E staining (10x), (**A**) follicular, (**B**) plexiform, (**C**) acanthomatous, (**D**) granular, and (**E**) desmoplastic. Analyzed by pathologist, Dr. Sherin James

**Fig. 2:**
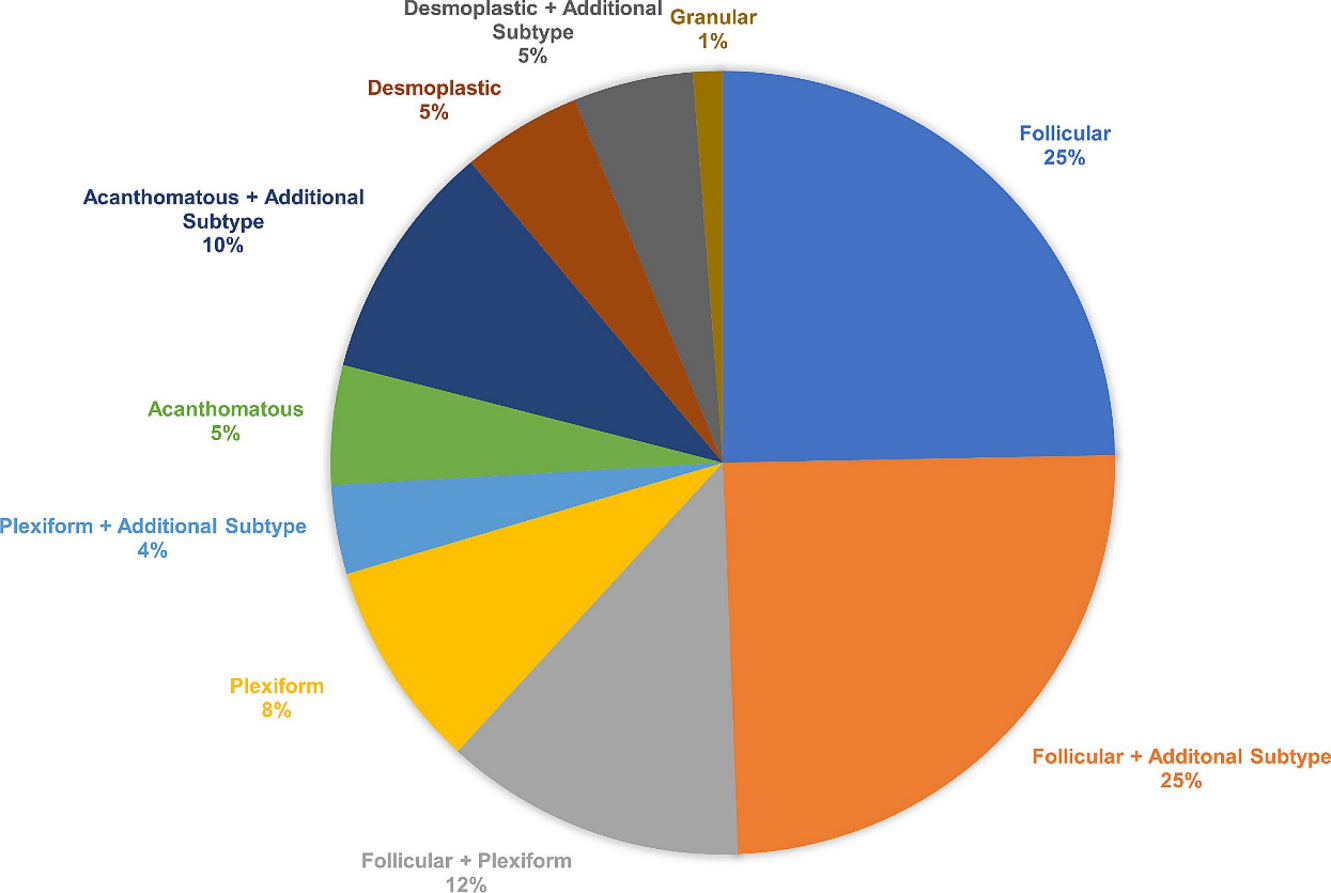
Frequency of histological subtypes in conventional ameloblastomas

Conventional ameloblastomas were subdivided based on histopathology: follicular, plexiform, acanthomatous, desmoplastic, or granular cell (Fig. 1). No samples were reported to have the basal cell type. Many tumors had a mixture of more than one pattern with the predominate patterns being follicular and plexiform. Of the wide number of histological subtypes found, the largest single pattern was follicular alone (15.5%) with an additional 15.5% including follicular as an additional subtype, followed by plexiform/follicular (7.8%), and plexiform alone (5.4%) with an additional 2.3% including plexiform as an additional subtype. Approximately 3.1% were acanthomatous alone with 6.2% including acanthomatous as an additional subtype. Desmoplastic included 3.1% alone and 3.1% including desmoplastic as an additional subtype. The granular subtype only encompassed 0.77% of specimens and was included with other histological subtypes. The major histological subtypes found in the patients reported with conventional ameloblastoma are shown in Fig. 2 (*n* = 52, 53.6%). The specific subtypes are summarized in Supplementary Table 1. The histological subtype was unknown or unrecorded for 46.4% of cases of conventional ameloblastoma (*n* = 45).

Unicystic ameloblastomas were subdivided into luminal (50%), intraluminal/plexiform (18.5%), or mural patterns (18.8%). In the pathologists reports the terms acanthomatous and desmoplastic were used to describe the histopathologic pattern of two cases of unicystic ameloblastoma (one of each pattern), which have been based on previous guidelines. Upon reexamination of the pre-surgical CT scans, the cases were confirmed to be unicystic. Histological specimens were unavailable.

### Comparison of Demographic Factors on Ameloblastoma Characteristics

An analysis was performed to compare the mean age and size of ameloblastomas by race (Tables 3 and 4). Table 3 presents the demographic breakdown of the cases by mean age. Direct comparison of the mean age of Black and White patients showed the mean age of White patients (47.8 ± 17.1 years) is significantly greater than Black patients (40.5 ± 18.9 years). When considering patients with primary ameloblastomas (i.e., with no documented recurrence), the mean age of White patients (48.9 ± 16.7 years) remained statistically greater than Black patients (40.2 ± 19.1 years), *p* = 0.017. For the size of the tumor, no statistical significance was found, with the mean tumor size of the White patients as 11.5 ± 13.2 cm^2^ and that of the Black patients as 16.1 ± 17.2 cm^2^, *p* = 0.229 (Table 4). There was a statistically significant correlation between age and racial groups, with *r* = -0.235, *p* = 0.011 (Table 3).

**Table 3:** Patient age distribution

Age (Average)	All	*P* value	Primary	*P* value	Recurrent		*P* value	
**All**	42.3 ± 18.73		42.2 ± 18.98		43.7 ± 15.9			
**White**	47.8 ± 17.1	0.038*	48.9 ± 16.7	0.017*	38.4 ± 49.7	0.396		
**Black**	40.5 ± 18.9		40.2 ± 19.1		50 ± 11.6			
**Male**	42.9 ± 17.7	0.717	43 ± 17.8	0.617	39 ± 18.4	0.669		
**Female**	41.6 ± 19.9		41.2 ± 20.4		45 ± 16.5			

Comparisons were age of racial group (Black vs. White) and sex (Female vs. Male) for primary and recurrent tumors using a two-group t-test

*P value < 0.05 considered significant

**Table 4:** Tumor size distribution

Size of tumor (Average)	All (cm^2^)	*P* value	Primary (cm^2^)	*P* value	Recurrent (cm^2^)	*P* value
**All**	14.80 ± 15.74		14.95 ± 16.04		12.8 ± 11.42	
**White**	11.8 ± 13	0.318	11.5 ± 13.2	0.229	14.5 ± 13.4	0.471
**Black**	15.7 ± 17		16.1 ± 17.2		6.6 ± 7.4	
**Male**	16.3 ± 18.2	0.643	15.9 ± 18.3	0.996	28.3 ± 5.3	0.059
**Female**	13.1 ± 12.5		13.8 ± 12.9		7.6 ± 7.1	

Comparisons were tumor size of racial group (Black vs. White) and sex (Female vs. Male) for primary and recurrent tumors using a two-group t-test

A comparison of the mean age and size of ameloblastomas by sex showed no statistical significance. The mean age of female patients (41.2 ± 20.4) was not significantly different from the mean age of male patients (43.0 ± 17.8), *p* = 0.617. The mean tumor size for female patients (13.8 ± 12.9 cm^2^) was also not significantly different from the mean tumor size of male patients (15.9 ± 18.3 cm^2^), *p* = 0.996. There was no statistically significant correlation between age and sex, with *r* = -0.067, *p* = 0.470. When this analysis was performed for only recurrent ameloblastomas, no statistical significance was found for race or sex (*p* > 0.05 for all results).

Multivariable analyses were performed to determine the difference in tumor size by race and age. Analysis was performed with age and race, or with age and sex, in the model for tumor size (as the outcome variable) across the ameloblastoma and recurrent ameloblastoma patients. No statistical significance (*p* > 0.10 for all tests on factors in each model) was found for any of the variables on tumor size, i.e., age and race were never jointly predictive of tumor size, and age and sex were never jointly predictive of tumor size.

## Discussion

Ameloblastomas are the most common odontogenic tumor in many parts of the world with a seemingly higher prevalence of ameloblastomas in sub-Saharan African and Asian populations [5]. Regezi, et al. (1978) showed that those of African descent had five times the likelihood of developing ameloblastoma when compared to Caucasians [25]. While previous research regarding the incidence of ameloblastomas has been conducted in regional sites in Africa and Asia with relatively homogenous demographics, there is still little analysis on the demographics of the patient population in a multi-racial area, like the United States [16–20]. In a study of ameloblastoma patients in South Africa, the patient distribution was 97.4% Black and 2.6% White [17]. Within the population of the southern United States, the demographics of the region are approximately 68.9% White, 26.8% Black, 4.4% Hispanic, and 1.9% Asian (Alabama Census). Data regarding ameloblastoma demographics has been sparse due to a low incidence rate across the population (odontogenic tumors as a whole have an incidence rate of 0.5 cases per 100,000 per year), coupled with patients being reluctant to seek medical care, or lacking access to care [4]. Incidence rates of 0.6 cases per 1 million inhabitants per year have been reported for Sweden compared to 5.6 cases in South Africa and Nigeria [3]. Thus, it has been a challenge to understand how demographics such as racial factors affect ameloblastomas. However, based on the patient demographics at our institution in the U.S. Southeastern region over a ten-year period, there was a higher prevalence of ameloblastoma in Black patients seen at our institution (61.3%). This agrees with the findings of higher prevalence in Afro-descendent patients found in regional sites (i.e., Nigeria, South Africa).

Our findings also agree with established literature of an earlier incidence of ameloblastoma [21, 26]. We found a statistically significant differences between the age of Black and White patients presenting with ameloblastomas. For ameloblastoma patients, the mean age of Black patients was 40.5 years and the mean age of White patients occurring at 47.8 years (*p* = 0.017, *n* = 129). While we did not find significance for size of ameloblastomas (*p* = 0.318), the mean tumor size trended larger in the Black patients (15.7 ± 17.0 cm^2^) compared to the White patients (11.8 ± 13.0 cm^2^). However, there is a large variance overall in the size of the tumors, as expected. Also, some patients may delay treatment due to the benign nature of ameloblastoma and the extensive surgery which may be recommended. More data is needed to clearly elucidate any relationship between the tumor size and race, as other factors may influence the size (such as time to discovery).

While the mean tumor size was larger in Black patients compared with the White Patients, the mean size of the recurrent tumor was much lower in Black patients compared to White patients (and female patients compared to male patients, respectively). However, given that there were only 9 recurring ameloblastomas, more data is necessary to draw any meaningful conclusion on the size of recurrent tumors across race or sex.

With respect to recurrence rate of 9 of the 129 total cases (7.0%), our rate is in agreement with the rate of 6.8% found in Nigeria where surgical resection was utilized as the primary treatment method [27]. In that study, no recurrence was found for cases where the resection margin was greater than 2.5 cm, and the smaller the resection margin the larger the recurrence rate, with 50% of the recurrent cases found for margins of 1.0 cm. Given that more conservative treatments (marsupialization, enucleation, curettage) have recurrence as high as 90%, segmental resection methods limit the recurrence to around 9% [18]. Thus, a combination of treatment option as well as the resection margins most likely led to our recurrence rate of 7.0%. In addition, it has been reported that recurrence is also highly related to other factors such as the presence of an impacted tooth, root resorption, cortical bone invasion, and soft tissue infiltration [19]. Further analysis is needed in how the treatment option, resection margin, and presence of other features affects the recurrence rate of ameloblastoma, especially in the differences among demographics. There is a possibility that there will be later recurrences with the slow tumor growth of ameloblastoma and lack of adequate follow-up, though this may be harder to elucidate given the length of study and patient tracking that would be required [2].

Apart from race predominance, literature so far has suggested that sex can be a large factor of predominance in some populations (e.g., Nigerian, Egyptian, Indian, and others), where male or females can have much higher prevalence of ameloblastomas, depending on where the study was conducted [2, 4, 17, 20–22]. Our study, like the results found by Santos, et al., found roughly an equal number of male and female patients (1.08:1 male to female), showing no overt predominance of either sex [20]. It may be possible there is another relationship between certain races having sexual predominance, but it was not apparent in our study, which was predominantly White and Black patients.

Ameloblastomas in our patient population were found mostly in the mandible (93.0%), which is consistent with findings from previous literature [20, 22–24]. Ameloblastoma subtype predominance also trended in the same direction, with most cases presenting as conventional, a minority of unicystic cases, and only one case of peripheral in our patient population. In addition, plexiform and follicular were also identified as the two most common histologic patterns, as was found in our data, and supported by prior work [4, 25]. A limitation of our study is that over the ten-year period, multiple pathologists had been consulted, some internal and some external to the UAB system. The naming conventions for ameloblastoma also changed over this time [7, 26, 27].

The clinical significance of the histopathology of ameloblastoma is contentious amongst the literature. Some reports state it has no significance, others state that plexiform or follicular patterns are associated with higher rates of recurrence or the expression of certain markers [28–34]. Gupta et al. reported plexiform ameloblastomas expressed significantly higher Ki-67 than follicular or acanthomatous types [31]. Higher levels of Ki-67 correlate with higher levels of cell proliferation and may correlate with a higher rate of tumor recurrence. Another study showed “abundant” expression of primary cilia, indicative of Hedgehog signaling, in follicular and plexiform types, which were rarely expressed in basal ameloblastomas [32]. Additional studies showed gene expression profile differences between plexiform and follicular ameloblastomas, with follicular ameloblastomas expressing variants of *BRAF*, *KMT2D*, and *ABL1*, while plexiform ameloblastomas expressed variants of *ALK*, *BRAF*, *KRAS*, *KMT2D*, *SMO*, *KMT2A*, and *BRCA2* [33, 34]. However, many of these studies did not consider ameloblastoma may show multiple histopathologies. It is possible with more ameloblastoma data or meta-data analysis the recurrence rate relationship on treatment type along with specific locations and ameloblastoma histology can also be determined in future works.

Based on work by Patel, et al. which systematically reviewed the ameloblastomas in Afro-descendants and non-Afro descendants, recurrent ameloblastomas accounted for 12% of the total study (in our study, recurrent cases accounted for roughly 7%) [35]. In addition, the BRAF V600E mutation is associated with up to 90% of ameloblastomas, and possibly related to recurrent tumors. However, a significant number of ameloblastomas are not genetically tested. The genetic testing of the BRAF V600E mutation in Black and mixed-race patients in the United States is worth future study, as the heterogeneity and genetic diversity of the population in the United States may shed additional insights into the role of BRAF V600E on primary and recurrent ameloblastomas, as well as lead to targeted non-surgical treatment options.

Typical patients of ameloblastoma present with only the symptom of slow-growing swelling. Consequently, patients generally tend to only seek medical advance when a deformity is evident, and sometimes patients may delay treatment due to the benign nature of the tumor but invasive surgical treatment. A critical missing piece is how factors such as time of discovery can impact the treatment and outcomes of patients. Thus, further data is needed to clearly elucidate any relationship between the tumor size and race, as factors such as time of discovery may impact the ultimate patient outcomes. Multi-variable analyses of the sex, age, and race did not reveal any statistically significant differences in the tumor size, although a limitation of our study was large standard deviations in tumor size. A larger sample size of patients may reveal trends, such as larger tumors in Black versus White patients, to be statistically significant. Another variable to consider is time to treatment, especially in areas such as the United States, where it may be variable among different demographics. Access to caer and socioeconomic status have been correlated with delayed diagnosis and poor health outcomes for many diseases, such as cancer and HIV, in the Southeastern U.S. [36–38].

In conclusion, we have shown the higher prevalence of ameloblastomas in Black patients within the southern United States over a ten-year period. Critically, we found significance between the age of Black and White patients, with Black patients presenting with ameloblastomas around 7 years earlier on average. While the size of the ameloblastomas between Black and White patients were not significantly different, we noted that the size of the Black patient’s ameloblastomas trended higher and should be studied further. Future ameloblastoma studies in the United States can incorporate genetic analysis, which may shed insight into how the BRAF V600E mutation can affect the development and recurrence of ameloblastomas in multi- and mixed-racial populations.

## Electronic Supplementary Material

Below is the link to the electronic supplementary material.

Supplementary Material 1

## Data Availability

Not applicable.
