# Supplementary material for: A Retrospective Analysis of 129 Ameloblastoma Cases: Clinical and Demographical Trends from a Single Institution
Source: J Racial Ethn Health Disparities. Author manuscript; Available in PMC 2025 Jun 1. (PMC11470111; doi:10.1007/s40615-024-01993-3)
Supplement: Supplementary file 1 [file NIHMS2007805-supplement-Supplementary_Table_1.docx]

**Supplemental Table 1: Histological subtypes recorded for conventional**

**ameloblastomas.**

| **Histological Subtype** | **All** | **Primary** | **Recurrent** |
| --- | --- | --- | --- |
| Follicular | 20 | 18 | 2 |
| Plexiform/Follicular | 10 | 10 | 0 |
| Plexiform | 7 | 7 | 0 |
| Mural | 6 | 5 | 1 |
| Luminal | 6 | 6 | 0 |
| Desmoplastic | 4 | 4 | 0 |
| Acanthomatous | 4 | 4 | 0 |
| Follicular/Acanthomatous | 3 | 2 | 1 |
| Follicular/Desmoplastic | 2 | 2 | 0 |
| Luminal/Mural | 2 | 2 | 0 |
| Follicular/Plexiform/Acanthomatous | 2 | 2 | 0 |
| Follicular/Desmoplastic/Acanthomatous | 1 | 1 | 0 |
| Acanthomatous/Desmoplastic | 1 | 1 | 0 |
| Follicular/Adenoid | 1 | 0 | 1 |
| Follicular/Granular | 1 | 1 | 0 |
| Plexiform/Acanthomatous | 1 | 1 | 0 |
| Unknown | 58 | 54 | 4 |
